# Biofilm Formation on Indwelling Medical Devices: A Review of Health and Economic Implications

**DOI:** 10.1155/ijm/3474643

**Published:** 2026-07-20

**Authors:** Gedif Meseret Abebe

**Affiliations:** ^1^ Department of Biology, College of Natural and Computational Science, Wolaita Sodo University, Wolaita Sodo, Ethiopia, wsu.edu.et

## Abstract

Indwelling medical devices represent an impressive stride in modern surgical practice and are widely used in procedures that support the body′s natural physiological functions. The global demand for these devices has surged, primarily driven by a range of clinical and demographic factors. However, their widespread use also carries risks, as foreign devices can introduce pathogens into the body during surgery and implantation. Shortly after implantation, host‐derived conditioning films rapidly coat the device surface, creating a foundation for bacterial attachment and subsequent biofilm formation. Biofilms are complex communities of microbial cells embedded within extracellular polysaccharide matrices on device surfaces. Bacteria within biofilms exhibit high resistance to antibiotics and host immune defenses, posing significant challenges for detection and diagnosis, largely due to the protective structural barrier and associated physiological changes. Biofilm‐associated device infections are persistent, chronic, and difficult to treat, often leading to treatment failure and recurrent infections. Recurring device‐related infections and repeated surgical interventions impose severe pain and substantial financial burdens on patients. Moreover, biofilms can induce device “corrosion,” compromising biocompatibility with surrounding tissues and shortening implant lifespan. Overall, bacterial attachment and biofilm formation on medical devices are notorious problems, causing device‐associated infections, device malfunction, and significant economic losses. Therefore, this review is aimed at providing an overview of biofilm formation on indwelling medical devices and its implications on health and economy.

## 1. Introduction

Rapid progress in surgical techniques, along with the development of advanced biomaterials, has profoundly transformed modern medical practice [1–3]. With the advent of biomaterials, various health defects have been treated, and saved lives of millions. Among the many breakthroughs in biomaterials, the discovery and utilization of indwelling medical devices represent an impressive stride in modern medicine and surgery [1, 4–7]. Recently, due to many driving factors, the demand for indwelling medical devices has surged worldwide [3, 8]. For instance, injuries represent a major global public health challenge, accounting for millions of deaths and disabilities each year [9]. Indwelling medical devices play a pivotal role in managing such trauma by providing essential life support and facilitating recovery [7, 10]. Similarly, in aging populations, there is a growing need to repair or replace soft and hard tissues such as bones, cartilage, blood vessels, or even entire organs [11]. Indwelling medical devices play an indispensable role in restoring or enhancing the function of these biological structures and systems [12]. Nowadays, a wide range of artificial body parts are used in modern medicine and surgery to replace/repair their natural counterparts [3]. Moreover, these devices have significantly improved the quality of life for patients suffering from a wide range of chronic diseases [3]. For example, millions of catheters are implanted annually to manage both acute and chronic conditions [13]. Generally, due to various clinical and demographic compelling factors, the demand for these devices has surged and become the mainstay of modern medicine and surgery.

Currently, tens of millions of medical devices, such as intravascular catheters, artificial heart valves, urinary catheters, breast implants, inflatable penile implants, implantable neurological stimulators, fracture‐fixation devices, dental and ossicular prostheses, and orthopedic implants, are utilized worldwide each year [10, 12]. However, the ever‐increasing use of such foreign devices in modern medicine can also introduce pathogens into the human body [14]. Although significant progress has been made in device design and surgical practice, nearly all medical devices remain vulnerable to microbial contamination originating from the patient′s flora, medical staff, inadequate handling, or environmental sources [7, 13, 15, 16]. Microbial contamination of indwelling devices persists as a critical challenge in contemporary surgical practice. Unless appropriate safety measures are implemented, surgical procedures and device implantation may provide opportunities for microorganisms to invade underlying tissues or colonize device surfaces, resulting in persistent infections and impaired device performance [3]. Even normal floras, which are harmless in their designated habitat, can pose significant health risks if they are introduced into unusual body sites or attached onto indwelling medical devices.

**Figure 1:**
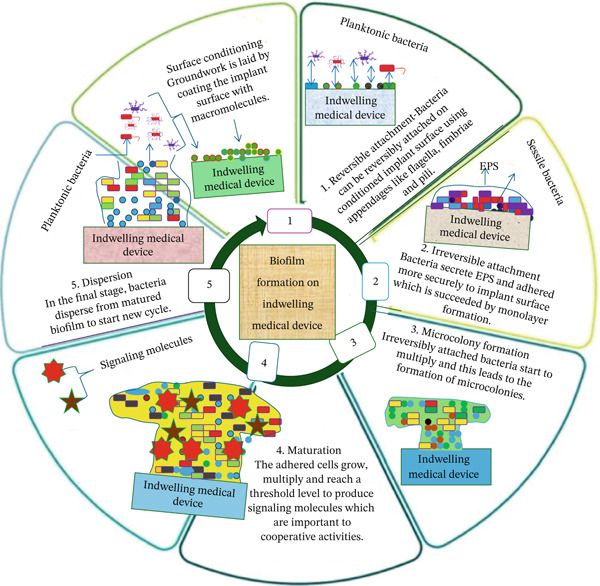
Biofilm formation on indwelling medical device [8, 17] with own modification.

**Figure 2:**
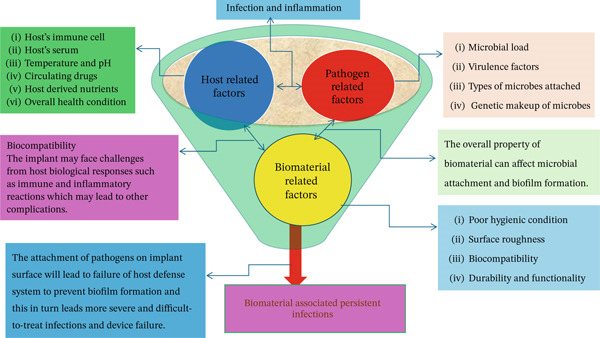
Biomaterial‐associated infection and factors that impact the rate and extent of biofilm formation on biomaterial [6, 19].

Once indwelling medical devices are implanted, bacterial attachment to its surface is not a straightforward process; rather, it involves complex interactions among the host, the device, and the microorganisms. Device lifespan, microbial attachment, and onset of infection depend on these physicochemical and biological interactions. Host‐derived conditioning films rapidly coat the surface of the device, laying the groundwork for bacterial adhesion and colonization (Figure 1). The rapid deposition of this film onto the surface of a medical device [18] presents a significant challenge, urging biomaterial scientists and engineers to explore novel alternative strategies to minimize or prevent microbial attachment on the surfaces of indwelling devices. So, understanding the interactions among host‐derived molecules, the device surface, immune responses, and microbial cells is crucial to design appropriate preventive mechanisms (Figure 2).

**Figure 3:**
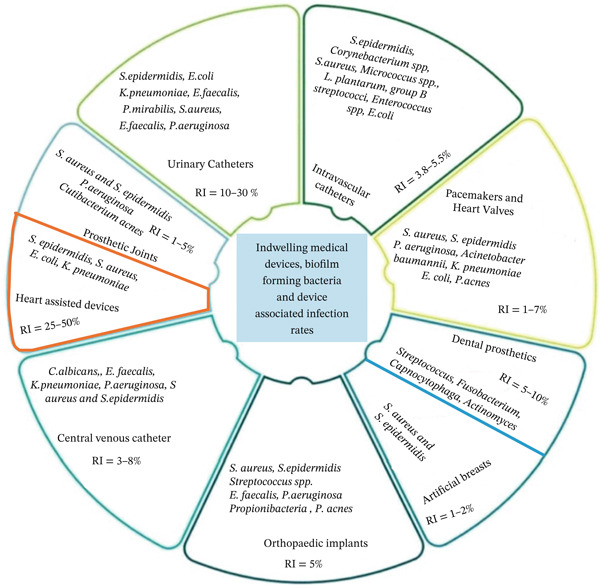
Medical indwelling devices and representative biofilm‐forming microorganisms and rate of infection (RI) [1, 24–34].

**Figure 4:**
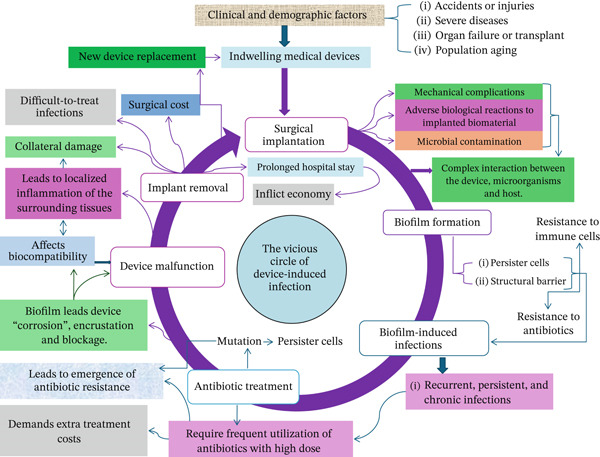
The vicious circle of device‐induced infection and implant removal, leading to revision surgery and recurrent infections [19] with own modification.

Bacteria can exist either in planktonic or sessile mode of life which enables them to adapt to and survive under varying environmental or host‐associated challenges. They secure their survival by forming structured microbial communities known as biofilms (Figure 1). These intricate microbial communities produce “glue‐like” extracellular polymeric substances (EPS), which facilitate adherence and maintain structural integrity [20–22] and protect bacterial cells within the biofilm from antibiotics and immune responses [23] (Figure 3). Biofilms are notoriously difficult to eradicate and exhibit high resistance to conventional antimicrobial therapies [35], primarily due to the “cement‐like” nature of EPS and the structural barrier they form. Biofilms are not only resistant to antibiotics and host immune defenses but are also extremely challenging to detect and diagnose using routine laboratory procedures [36]. Apart from this, the growth rate and physiology of bacteria within biofilms differ significantly from their planktonic counterparts. Biofilm‐forming bacteria can generate dormant persister cells, which are metabolically inactive and highly resistant to both antibiotics and immune clearance [37, 38] (Figure 4) and pose significant challenges for detection and diagnosis [36]. When conditions are conducive enough, persister cells can revert to an active state, leading to the recurrence of infection and treatment failure [38, 39]. These physiological adaptations can also accelerate the development of multidrug resistance (MDR) in biofilm‐forming bacteria. In this communal mode of life, bacteria exhibit significantly higher rates of horizontal gene transfer [40], primarily due to the close proximity of cells within the biofilm matrix and the long retention time of cells within the EPS matrix [23] (Figures 1 and 4). This in turn facilitates the exchange of antibiotic‐resistance genes, contributing to the emergence and persistence of MDR bacteria in medical settings [41]. Even the benign normal flora can get antibiotic resistance genes from MDR bacteria and become major human pathogens [23]. Nowadays, the prevalence of antibiotic resistance has steadily increased [42] and is recognized as one of the most pressing public health threats worldwide, driven by multiple factors including biofilms [43]. Bacterial cells embedded within the biofilm matrix are also highly prone to mutations that enable them to develop resistance [37, 42], allowing them to adapt and survive under hostile conditions [44]. Following such stressful growth conditions, surface‐attached microbial cells can detach from the device, revert to a planktonic mode of life, and disseminate to other sterile sites [45]. Therefore, biofilm‐contaminated devices can serve as bacterial reservoirs, releasing planktonic cells that disseminate to distant body sites and cause secondary infections.

Microbial contamination of sterile devices and biofilm formation is an inevitable event [46], posing significant risks to patients. Biofilm‐forming bacteria cause a broad spectrum of infections, including device‐associated infections, which constitute some of the most serious complications in modern medical practice [47], with significant economic consequences. Because of their inherent tolerance and resistance of biofilms to antimicrobial treatments and host immune system, biofilms give rise to recurrent, persistent, and chronic infections that are often difficult to treat and eradicate. As noted previously, arrays of indwelling devices are widely used in clinical treatments; however, they are also prone to bacterial colonization and biofilm formation, becoming foci of device‐related infections that often require device removal and result in costly treatments [10, 48, 49]. The susceptibility of these devices to bacterial attachment and colonization largely accounts for the rise in device‐associated infections. Recent research has revealed a surge in nosocomial infections, largely attributed to the use of biomaterials such as heart valves, artificial veins, joint prostheses, orthopedic implants, and urinary tract catheters [50]. Moreover, many of the most serious bacterial diseases affecting humans today are caused by bacterial biofilms that produce chronic diseases [51]. For instance, 65% of nosocomial infections, 80% of chronic infections [52], and more than 80% of bacterial infections [53] including endocarditis, osteomyelitis, cystic fibrosis, and chronic sinusitis [54] are caused by mainly biofilm‐forming bacteria. Research reports estimate that up to 1 million deaths each year are attributed to biofilm‐associated infections [43].

**Figure 5:**
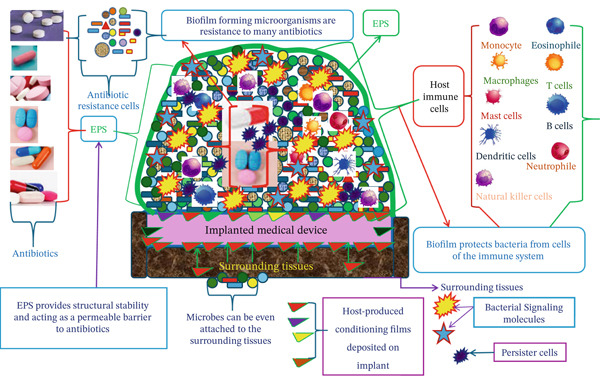
Biofilm formation on indwelling medical devices and its interaction with antibiotics and cells of the immune system.

Contamination of medical devices and subsequent biofilm formation not only causes hospital‐acquired infections [55] but also compromise device functionality [17, 24, 56]. Biofilm‐induced implant‐associated infections are the challenging problems in public health that require mostly both device removal and extended antimicrobial treatment [57]. They are a multifactorial health problem arising from complex interactions among the implanted device, the host′s immune system, and microbial contaminants. For instance, indwelling medical devices represent a site of competition between the integration of the material into surrounding tissues and the adhesion of bacteria to the implant surface [58] (Figure 5), as well as the host immune response. In these competitions, the implanted device can serve as a supportive substratum for bacterial attachment and biofilm formation [59, 60], which is a major factor in the pathogenesis of device‐induced infections, device malfunction, and eventual removal [25, 56]. Bacterial deposition on the surfaces of indwelling medical devices, along with host‐derived surface coatings, can also lead to device “corrosion,” thereby compromising biocompatibility with surrounding tissues and shortening the lifespan of the devices. For instance, urethral catheters can be colonized by urease‐producing species such as *Proteus mirabilis*, which form unusual crystalline biofilms that encrust catheter surfaces, block urine flow, and reduce the lifespan of implanted devices [61]. In addition, device‐associated infections trigger local tissue responses, leading to acute and chronic inflammation, foreign body reactions, granulation tissue formation, and fibrous encapsulation, all of which further compromise device function and patient health [62]. As a result, device removal is inevitable, requiring difficult and expensive interventions [63]. Moreover, repeated surgical procedures can have collateral damage (Figure 4), thereby increasing further health complications.

Healthcare‐associated infections are major health and economic problems that affect both developing and developed regions of the world [64]. In particular, in developing countries, including those in sub‐Saharan Africa where hygiene standards are often inadequate and financial constraints are prevalent, healthcare‐associated infections impose a substantial health and economic burden on societies [65]. Although these infections can also arise from planktonic microorganisms [64], biofilm‐induced infections impose a significant global economic burden on society [66]. The global economic burden attributed to biofilm‐related infections is estimated to be approximately $5000 billion per year [23]. Biofilm‐induced device‐associated infections often necessitate intermittent treatments, surgical interventions, and frequent device replacements, which are costly medical procedures that impose significant financial burdens on patients [67–69]. From both health and economic perspectives, such infections are perilous to patients, demanding substantial treatment costs. Overall, biofilm‐associated infections lead to increased patient morbidity and mortality, prolonged hospital stays, frequent device replacements, and significantly higher healthcare costs.

The ubiquity of microorganisms and the vulnerability of devices to contamination pose major challenges, frequently leading to device‐related infections with significant morbidity and mortality [15, 70]. Despite improvements in hygiene conditions and sterilization measures in hospitals [3, 7], contamination of indwelling medical devices remains an inevitable problem [46] that impairs device function and leads to severe, difficult‐to‐treat infections. This is mainly attributed to biofilms which are highly resistant to cleaning, disinfection, and conventional sterilization processes. Biofilms exhibit remarkable tolerance to antimicrobial agents and host immune defenses, making eradication of the infection without device removal nearly impossible [71]. To date, no therapeutic agents have been approved for clinical use that specifically target bacterial biofilms [72, 73]. This persistent challenge underscores the urgency of the problem and highlights the need for novel prophylactic and therapeutic strategies [74]. Due to the recalcitrant nature of biofilms, surgical excision of infected implants and wound debridement are the primary treatment approaches practiced in clinical area. Unfortunately, these procedures are frequently associated with considerable physical trauma and a high risk of severe complications [75]. Especially in developing countries, where poor hygiene conditions, limited resources, and weak healthcare infrastructure are prevalent, such complications impose a substantial burden on patients.

Once bacteria attach to the surface of a device, they become extremely difficult to eliminate, leading to chronic and persistent infections. The implementation of effective preventive strategies is imperative to inhibit bacterial adhesion and subsequent biofilm formation at the earliest stages. Disrupting quorum sensing (QS), preventing bacterial attachment, inhibiting aggregation, degrading EPS, and targeting persistent cells through nanoparticle‐based antimicrobial complexes are key strategies currently being explored to combat biofilm‐related infections [66]. For instance, in recent years, nanoparticles have emerged as a promising strategy for combating biofilms [76] or treating pathogenic biofilms on indwelling medical devices and implants [77]. Phage therapy has also re‐emerged as a therapeutic approach that selectively targets antibiotic‐resistant bacterial strains while exerting minimal effects on the normal microbiota [78]. Although these promising research findings are impressive strides in realms of modern medicine, biofilm‐induced device‐associated infections continue to challenge public health, impair device performance, and impose substantial economic burdens. This underscores the need to explore all potential avenues for developing novel strategies to prevent or inhibit biofilm formation on indwelling medical devices in practice. Therefore, this review article is intended to give a highlight on biofilm formation on indwelling medical device and its implications on health and economy.

## 2. Steps of Biofilm Development on Indwelling Medical Devices

In order to prevent biofilm‐induced infections or develop antibiofilm treatments and therapies, first and foremost it is better to understand the basic steps of biofilm formation on different surfaces. The adage “prevention is better than cure” works for biofilm‐induced infections. Once microorganisms organize into a structured biofilm, they develop protective mechanisms that make them highly resistant to both antibiotics and immune response.

Recurrent infection is their prominent feature which leads to severe complications and other collateral damages to nearby tissues and distant sites. Therefore, it is better to have some basic information on how microbes built their protective structures, which cellular structures are involved in building these structures, how they cooperate and communicate with each other, how they regulate biofilm formation, and so on. These are the most important target sites, in order to prevent microbial attachment and biofilm formation on different surfaces including indwelling medical devices.

Biofilms can be attached to different biotic and abiotic surfaces and consists of a complex aggregation of microorganisms that are surrounded by self‐produced matrix of EPS. This communal life is the most successful and prevalent lifestyle where resistance to hostile conditions such as antibiotics and the immune system is their prominent feature [79, 80]. The process of biofilm formation consists of many steps, starting with attachment to a living or nonliving surface that will lead to the formation of a microcolony, giving rise to three‐dimensional structures and ending up, after maturation, with detachment [79, 81, 82] (Figure 1). The transition of a bacterium from a planktonic mode of life to sessile cells is a multistage and regulated process that depends on several factors [83] such as bacterial cell surface, substratum, and growth media [84, 85] (Figure 1). Pathogens induce a disease process by forming organized biofilms that enable them to attach, cooperate, replicate to accumulate, and express their virulence potential [86]. Biofilm‐forming bacteria can be regarded as a social “enterprise,” in which individual cells coordinate their activities to build protective structures and collectively withstand adverse environmental stresses [63] (Figure 5).

### 2.1. Conditioning Layer

The process of microbial colonization and biofilm formation starts by the conditioning of the surface (Figure 1). Surface conditioning is a stepping stone to attachment and paves the way for microbes to establish structured biofilm communities. Once medical devices are implanted, surface conditioning will follow instantaneously [25]. For instance, if a surface is immersed in an aqueous medium, it will be coated with polymers and form thin films; this in turn has a profound impact on the rate and extent of microbial attachment [26] (Figure 1). This thin layer of conditioning films deposited on indwelling medical devices comprises proteins such as fibronectin, fibrinogen, vitronectin, thrombospondin, laminin, collagen, and polysaccharides [8] (Figure 5). Host‐derived conditioning films from blood, tears, urine, saliva, intervascular fluid, and respiratory secretions significantly affect the adherence of bacteria to indwelling medical devices [26]. These polymeric substances which coat the implanted device may not be similar, and they depend on the site where the implant is found. For instance, for venous implants, surface coating is facilitated by organic macromolecules such as pyruvate, glucose, and fibrinogen, whereas for urinary implants, proteins, electrolytes, and other organic molecules are responsible for film formation [25]. Generally, these conditioning films create a favorable substratum for bacterial attachment and subsequent colonization on the surfaces of indwelling medical devices.

### 2.2. Reversible Attachment

This is the critical step where microbes will start interacting with different surfaces [27]. In this step, weak forces such as van der Waals and electrostatic forces are involved for initial reversible attachment [6, 26]. Using either physical forces or appendages such as pili, fimbriae, or flagella, planktonic cells come in contact with conditioned surfaces [8] (Figure 1). Depending on this interaction and other environmental cues, cells may become either sessile, or they may detach from the surface and reverse back to the planktonic phase [85] (Figure 1). In order to avert further biofilm development, antibiofilm treatments and therapies should be given at its infant stage [87, 88]. Once microbes proceed to the next phase of biofilm development, they will start undergoing both structural and physiological modification and thereby secure their survival under any hostile conditions such as antibiotics and immune cell response.

### 2.3. Irreversible Attachment

During biofilm formation microbial cells can switch from loosely reversible attachment to a more stable irreversible attachment, which is greatly influenced by adhesive structures of bacteria such as flagella, pili, fimbriae, and glycocalyx, and these appendages enable bacteria to attach firmly on surfaces and resist repulsive forces [17] (Figure 1). Along with these adhesive structures, short‐range interactions such as hydrophobic and dipole–dipole interactions, ionic, hydrogen, and covalent bonding play a pivotal role in irreversible attachment [89]. Based on these interactions, reversibly attached cells become irreversibly attached when the attractive force is greater than repulsive forces [8], and then these irreversibly attached bacteria will commence multiplication and form microcolonies [17] (Figure 1), which can be considered as a basic unit of mature biofilm [26].

### 2.4. Microcolony Formation

Bacterial cell multiplication and intercellular adhesion takes place when they have attached themselves to the implant′s surface [24]. Since biofilm formation is collaborative microbial work, cells must be irreversibly attached and proliferate in number and form microcolonies. In order to transit from single cells to microcolony communities, multicellular behavior must be coordinated. For instance, bacteria secrete signaling molecules into the surrounding cells that enable them to start the process of forming the microcolony structure [90], which is a thin layer at its early stages. But through time, they begin to produce EPS, which accounts for the majority of the dry biomass of mature biofilm [85, 91] (Figure 5). The EPS provides mechanical stability for the three‐dimensional microorganisms and binds them to the surface [92]. EPS acts as an umbrella, and under this umbrella, a complex group of microbial cells are adhered to this matrix and present on the surface of medical devices [24] (Figure 1).

### 2.5. Maturation

Bacterial cells become more “armed” by undergoing both structural and physiological changes. Beside structural and physiological changes, cells can also develop signaling language that can facilitate their cooperative activities. The adhered cells grow and mature by interacting among themselves, and produce auto‐inducer signals which result in the expression of biofilm specific genes [8]. This complex intra‐ and intercellular communication is based on a signaling system regulated by QS and implicated in the regulation of very different and complex physiological processes, depending on cellular density [93, 94] (Figure 5). This signaling “language” enables bacteria to intercommunicate and collaborate to survive in even the hardest conditions [95]. Attachment of the bacteria to a surface results in the rapid alteration in the expression of a number of genes responsible for EPS or “slime” production and maturation [96]. In this stage, the bacterial cells start secretion of the EPS that encloses the cells, thus stabilizing the biofilm network and protecting themselves from antibacterial agents [8]. Some species of bacteria generate instinct structures within the biofilm, such as the mushroom cap that initiates under certain conditions [90] (Figure 1).

### 2.6. Dispersion

Surface attached microbial cells are not attached to surfaces once and for all. Depending on the environmental cues they encounter, microorganisms can alternate between sessile and planktonic modes of life (Figure 1). Dispersion enables bacteria to spread from one region of the body to another, thereby spreading infection and posing a significant threat to the host [8, 25]. Environmental cues such as changes in temperature, starvation, oxygen deficiency, or metabolite accumulation can trigger planktonic cells to disperse from the mature biofilm [85]. Moreover, cells can also manage dispersal activity by upregulating and downregulating genes required for cell motility, such as flagella synthesis, EPS degradation, and genes essential for adhesive structure production, including EPS, pili, and fimbriae, respectively [89]. Additionally, QS molecules such as acylhomoserine lactones, diffusible fatty acids, and peptides can influence the rate of dispersion [36]. Generally, microorganisms attached to the surfaces of medical devices enter into a biofilm state in which they display unique growth rates, structural and physiological changes, and resistance against antimicrobial agents and host immune mechanisms compared with their planktonic counterparts [27, 97].

## 3. Health Implication of Device‐Associated Biofilms

Indwelling medical devices play a prominent role in modern medical and surgical practices. As noted previously, tens of millions of devices are implanted each year to replace any living tissue that has undergone some accidental damage or destruction [58, 63, 98]. Based on the severity of the problem, these devices can be used intermittently, for months, years, or permanently to ameliorate the suffering of many patients [58, 99]. Despite their indispensable role in surgical practices, indwelling device‐related infections are the most serious and devastating complications associated with the use of biomaterials [83]. These infections are a multifactorial process that involves the interaction between host, indwelling device, microorganisms, and their by‐products [100] (Figure 2). Bacterial adhesion and biofilm formation on the surfaces of medical devices is significantly influenced by physical and chemical properties of the medical material, body fluids surrounding the device, nutrient, host environment, the microbial load contaminating the device, and temperature [85, 101] (Figure 2). The nutrient available on implant surface provides an optimum environment for microbial attachment and biofilm formation which can increase likelihood of survival and potential for symbiotic relationships [102]. Different implant‐associated infections can be presented depending on the balance of these contributing factors [100]. Therefore, implant infection involves complex interactions between the pathogen, the biomaterial, and the host immune response [48] (Figure 2).

Currently, different medical devices such as intravenous catheters, prosthetic heart valves, joint prostheses, orthopedic fixation hardware, peritoneal dialysis catheters, cardiac pacemakers, cerebrospinal fluid (CSF) shunts, and endotracheal tubes save the lives of millions [22, 28, 45]. But biofilm formation on these indwelling devices is difficult to thwart and poses an enduring problem on public health and devices. Contaminated indwelling devices can cause severe complications such as reoperation, which can prolong the recovery period and thereby increase the suffering of patients. Bacterial infections are among the most severe complications, mostly caused by the contamination of implantable biomedical devices [22], linked to biofilm formation and subsequent infection [103]. The surface of inserted medical devices or other implants in the body, such as catheters, artificial joints, heart valves, pacemakers, breast implants, contact lenses, and ventilation tubes, is prone to contamination and subsequently biofilm formation [45]. The majority of device‐associated infections, like cystitis, catheter‐related sepsis, and endocarditis, can be attributed to bacteria adhered to these medical devices [104] (Figure 3). Even if the severity of the problem varies with device type, invasiveness, anatomic insertion site, and duration of use (temporary, short‐term, long‐term, or permanent), implant infections are the most frequent and severe complications associated with the use of biomaterials [48]. For instance, long‐term medical implants can cause inflammation and tissue destruction around implants; sometimes, these infections are life threatening [105].

Bacterial biofilms are known to play a tremendous role in the pathophysiology of many infectious diseases, including device‐associated infections [106]. Biofilm‐associated infections are one of the major threats of modern medicine. As research reports indicated, the majority of bacterial infections are biofilm‐induced. For instance, 60%–85% of all microbial infections involve biofilms developed on natural tissues or artificial devices such as central venous, peritoneal, and urinary catheters, dental materials, cardiac valves, intrauterine contraceptive devices, contact lenses, and implants [93, 107–109] (Figure 3). Prolonged stay in hospital, chronic infection, morbidity, mortality, device replacement, and subsequent reimplantation and resistance to antibiotics and immune cells are the prominent features of biofilm‐induced infections [10, 110].

Based on the type and location of implants, duration and other contributing factors, microbes can cause different kinds of biofilm‐induced infections. As reports revealed, different kinds of device‐associated infections have been documented for different medical devices [82]. Different device‐related infections such as breast implant (2%), joint prostheses (2%), pacemakers and defibrillator (4%), mechanical heart valve (4%), ventricular shunts (10%), and ventricular devices (40%) infections are attributed to biofilm‐induced infections [17]. Different medical device–induced infections and infection rates are reported which are mainly attributed to biofilm formation on these implants (Figure 3).

As many research reports revealed, attachment and biofilm formation on different surfaces is an inevitable microbial survival strategy on different hostile conditions. As shown in Figure 3, a plethora of bacteria and fungi form biofilm on surfaces of different implants and cause infections. Depending on the type of the implant and on the anatomical site of implantation, a wide range of bacterial species such as *Enterococcus faecalis*, *Staphylococcus aureus*, *Staphylococcus epidermidis*, *Streptococcus viridans*, *Escherichia coli*, *Klebsiella pneumoniae*, *P*. *mirabilis*, and *Pseudomonas aeruginosa* can form biofilm on medical devices and cause implant infections [8, 48] (Figure 3). For instance, coagulase‐negative *S*. *epidermidis* and *S*. *aureus* are commonly reported infection‐causing agents related to indwelling medical devices such as vascular catheters, prosthetic joints, and artificial heart valves [111, 112] (Figure 3).

Both *S*. *epidermidis and S*. *aureus* are estimated to cause about 40%–50% of prosthetic heart valve infections, 50%–70% of catheter biofilm infections, and 87% of bloodstream infections [8]. Besides biofilm formation on the surface of tissues and organs or implants, *S. aureus* can also form persister cells within biofilm which are recalcitrant to hostile conditions and thereby cause chronic infections that are nonhealing [113]. Similarly, the biofilms of *P*. *aeruginosa* in the lungs of patients with cystic fibrosis, and dental‐plaque biofilms produced by several *Streptococcus* species are the prominent examples of chronic infections [114]. For instance, biofilm‐forming *P. aeruginosa* is resistant to the host immune system and antibiotic treatment, and it is the main cause of morbidity and mortality [115].

Biofilm‐forming microbes attached to medical devices are difficult to remove from their surfaces, and they need prolonged hospitalization, surgery, and long‐term antimicrobial treatment, which are unaffordable in terms of cost [27]. Removal of the colonized device or surgical excision of infected tissue is the only efficient way to eradicate a biofilm‐related infection [116]. Similarly, other reports also support surgical excision of the implant and local debridement, but the process is expensive and can cause socioeconomic problems for patients and it can also entail a risk of recurrence [117] (Figure 4). Additionally, collateral tissue damage and recurrent microbial infections are enduring problems in device‐associated infections. Treatment of device‐induced infection is a vicious circle where the problem is not solved once and for all [19] (Figure 4). Beside health implications, biofilms can cause encrustation and blockage that may ultimately interfere with implant function, compatibility, durability and performance [24, 31, 100] (Figure 2). This leads to the failure of the device, replacement of the medical implant, or a second surgery, resulting in a significant increase in mortality, treatment costs, and recovery time [45] (Figure 5). Generally, biofilm formation on the surface of indwelling medical devices is a multifactorial problem that paves the way for the emergence of antibiotic resistance and host immune evasion, causing serious threats to patient health and impairment of device function.

### 3.1. Biofilm and Its Interaction With Antibiotics and Host Immune Cells

Biofilms have unique characteristics that can determine their interaction with antibiotics, host immune cells, and other hostile environmental conditions. Biofilm formation on indwelling medical devices plays a critical role in the emergence of antibiotic resistance. Since bacteria within biofilms are very close to each other, they exchange their QS molecules, resistance genes easily, and show heterogeneous character in each biofilm community [118] (Figure 5). Bacterial biofilms are just like “marketplace” where bacteria can exchange “goods” for their daily survival including nutrients, instructions, resistance genes, and “weapons.” Besides providing a protective matrix, biofilms are also ideal sites for horizontal gene transfer and enable bacterial survival in hostile environments [119]. Bacteria embedded within biofilms are structurally and physiologically “armed” to resist any pressure coming from antimicrobials and soldiers of the immune system (Figure 5).

Biofilm‐induced device‐related infection and the emergence of antibiotic resistance pose a considerable health problem and financial burden on healthcare systems [120, 121]. The formation of bacterial biofilm is one of the main contributors to the emergence of MDR that makes bacterial infection a major threat to public health and the economy [122]. The enormity of the problem is more severe for public health if MDR pathogens can form biofilms [123]. Currently, the emergence of MDR bacteria is a serious health issue that threatens public health, including medical devices [46]. The formation of biofilms on the surface of biomaterials and the development of antibiotic‐resistant bacteria have reduced the effectiveness of conventional antibiotic treatment of infections [124]. Biofilm‐forming bacteria are difficult to control using antimicrobial agents as a consequence of intrinsic and acquired resistance mechanisms [33]. Medical device–associated biofilms facilitate recalcitrant or recurrent infections despite the use of appropriate antibiotics [112] (Figure 4). This is because the biofilm structures can compromise the human defenses and prepare a shelter for microorganisms, leading to immune evasion, bacterial persistence, drug tolerance, and resistance [33, 91, 125] (Figure 5). As indicated in Figure 5, biofilms can serve as a protective refuge for bacteria since the extracellular biofilm matrix can act as a barrier preventing the infiltration of the community by hostile conditions [126], such as antibiotics and the host′s immune cells [21]. As research reports revealed, the resistance of bacteria in biofilms to antibiotics can be 10–1000 times that of the corresponding planktonic cells [105]. This increased resistance can be attributed to different factors, including decreased diffusion of antimicrobial agents through the self‐produced extracellular matrix, altered metabolic activity, and formation of persister cells [95] (Figures 4 and 5). Biofilm‐induced infections require a dose of antibiotics, but the overuse of antibiotics paves the way to emergence of resistant microorganisms [127], which further worsens the problem. Under such stressful conditions, bacteria can undergo mutations and develop resistance genes in response to prolonged antibiotic exposure (Figures 4 and 5).

Biofilm‐forming cells undergo phenotypic shift and become persistent to antibiotics and host immune responses. Biofilm‐forming bacterial cells grow slowly, so that the majority of antibiotics are ineffective in inhibiting or killing persister cells, and they are also resistant to the immune system [128]. Biofilm‐residing bacteria can be resilient to the immune system, antibiotics, and other treatments. Because of their structural and physiological changes and adaptations, bacterial cells attached to indwelling medical devices exhibit high tolerance to antimicrobial agents and host immune defenses (Figure 5), making the treatment of such infections nearly impossible without device removal. These protected modes of growth on medical devices impede host defense mechanisms, and hence, microbial cells could be out of immunosurveillance systems. As shown in Figure 5, immune cells, such as dendritic cells, natural killer cells, neutrophils, macrophages, and monocytes, utilize pattern recognition receptors (PRRs). These PRR molecules play a critical role in recognizing pathogen‐associated molecular patterns (PAMPs) and thus distinguish self from invading microbes. But in a biofilm, the majority of common PAMPs are concealed by EPS and other external biofilm structures [129]. Besides this, pathogens undergo structural modification so that they will be out of immune surveillance radars. During a biofilm infection, both innate and acquired host immune responses might be activated simultaneously; but neither of which are able to eliminate the biofilm pathogen, instead accelerate collateral tissue damage [108] (Figure 4). The interaction between the biofilm‐forming bacterial cells and soldiers of the immune system results in collateral damage of adjacent tissues which is an important aspect of biofilm infection pathology [128] (Figures 4 and 5).

Biofilm‐associated infections are difficult to treat for numerous reasons including strong physical adherence, high antibiotic tolerance, immune evasion, and immune response antagonism [108] (Figure 5). For instance, treatment of implant‐associated infections includes delivery of high dose antibiotics and/or replacement of the implant involving costly and risky surgeries, both of which are ineffective due to antibiotic‐resistant strains and high chances of reinfection on the new implant [8]. Antimicrobial therapy fails without removal of the implanted device [111]. It is hard to treat such infections without removing the device because of their high tolerance to antimicrobial drugs and host defense mechanisms (Figure 4). Generally, microbes attached on the surfaces of medical device undergo both structural and physiological change so that they are resistant to antibiotics and host immune cells.

## 4. Economic Implication of Device‐Associated Biofilms

**Figure 6:**
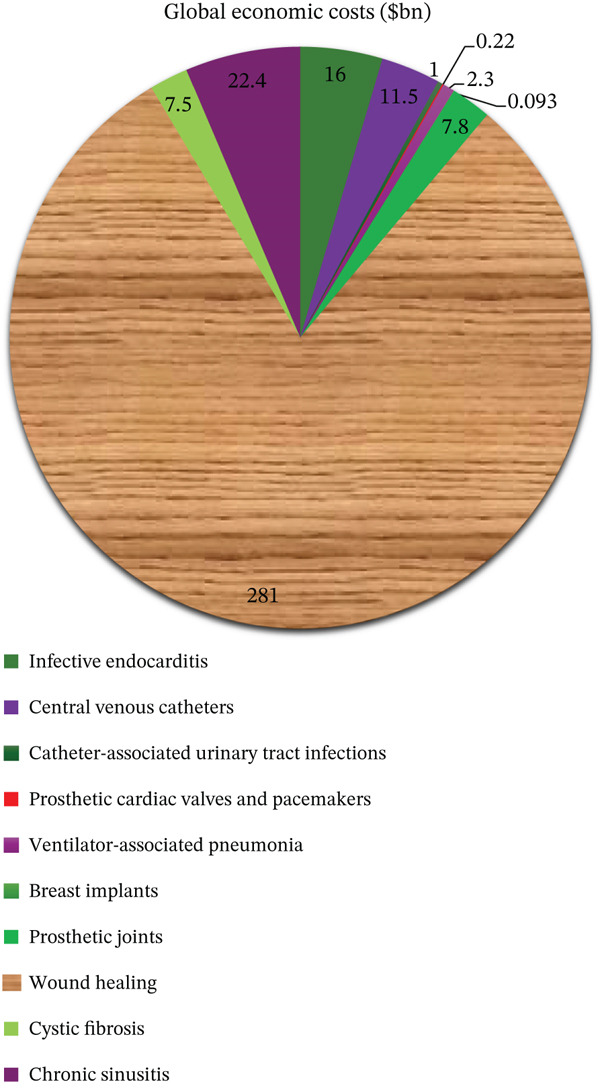
A glimpse of a global economic implication of biofilm‐induced infections [44].

Besides causing severe health complications, biofilm‐induced device‐associated infections have far‐reaching economic impacts (Figure 6). Biofilm‐driven infections are typically characterized as recurrent, chronic, or persistent, leading to prolonged hospital stays and increased treatment costs. These infections are seldom eradicated completely, necessitating repeated hospital visits for ongoing follow‐up care, additional treatment, or device replacement, thereby contributing to a significant financial burden. Biofilm‐driven infections become a significant socioeconomic burden for patients by causing hospital stays, patient suffering, reduced life quality, increased mortality risk, and lost employment [128]. The other economic burden that comes along with frequent hospital visits is that transport, food, and other related costs.

Nosocomial infections are an overarching issue in modern medicine affecting public health, economy, and medical devices. Incessant microbial contaminations of medical devices become an enduring problem in healthcare settings that lead to life‐threatening infections and inflict economic damage. Currently, implant devices account for approximately 45% of all nosocomial infections and scourge both public health and the economy. In the United States, for example, approximately 2 million nosocomial infections cost nearly $11 billion annually [4]. The majority of these nosocomial infections are attributed to microbial biofilms attached to both biotic and abiotic surfaces. As the medical sector is progressing, the use of indwelling devices is increasing both in numbers and in variety [67]. The global production of biomedical devices and tissue engineering‐related materials is a $180 billion per year industry and expanding rapidly. In the United States alone, over 5 million medical devices or implants are used per annum [28]. For instance, more than 5 million central venous catheters are implanted annually in the United States, of which more than 80,000 lead to catheter‐related bacteremia [130]. Similarly, in Germany alone, more than 2.5 million biomedical devices such as central venous catheters, prosthetic joints, cardiac pacemakers, heart valves, artificial lenses, and CSF shunts are used annually [111]. However, these devices are susceptible to microbial colonization and infection that can lead to health complications and economic [28].

The prices of biomedical devices are increasing from year to year globally, which imperil both the economic and health sector. As reports indicated the estimated usage of medical polymers is set to keep rising with the 2017 US market valued at ca. $5 billion and forecasted to rise to ca. $7 billion by 2020 [25]. The current figure regarding the expense may exceed the abovementioned costs. Along with other factors, microbial biofilm formed on the surface of indwelling medical devices is one of the critical contributing factors in an increment of the price of these devices. As research reports revealed, the global annual economic burden of biofilms is estimated to exceed $5 trillion [131]. Biofilm formed on medical implants causes a number of microbial infections and approximately $3790 million amount globally per year is spent on treating and diagnosing biofilm‐related catheter‐associated urinary tract infections [17] (Figure 6). In the United States, more than 500,000 biofilm‐related implant infections occur annually. Among these, prosthetic joint infections alone are projected to generate revision surgery costs exceeding $500 million per year [54].

Biofilm‐induced device‐associated infections are persistent and chronic, often necessitating frequent device replacement, prolonged hospitalization, and the administration of high doses of antibiotics, all of which impose substantial economic burdens. Biofilm‐induced device‐associated infections are the main economic burden for developing nations. For instance, prosthetic medical devices are risk factors for chronic infections in developed countries, and these infections are characterized by slow onset, middle intensity symptoms, chronic evolution, and resistance to antibiotic treatment [93]. These device‐associated infections can cause major medical and economic sequelae [132]. The economic cost of biofilm‐induced device‐associated infections could be higher in developing countries. However, assessment of the economic burden of biofilm‐induced infections in developing countries is hindered by the scarcity of up‐to‐date and comprehensive evidence. Generally, device‐related biofilm infections increase hospital stays and add extra hospitalization costs which could have economic implications for patients.

## 5. Prevention of Biofilm Formation on Indwelling Medical Devices

**Figure 7:**
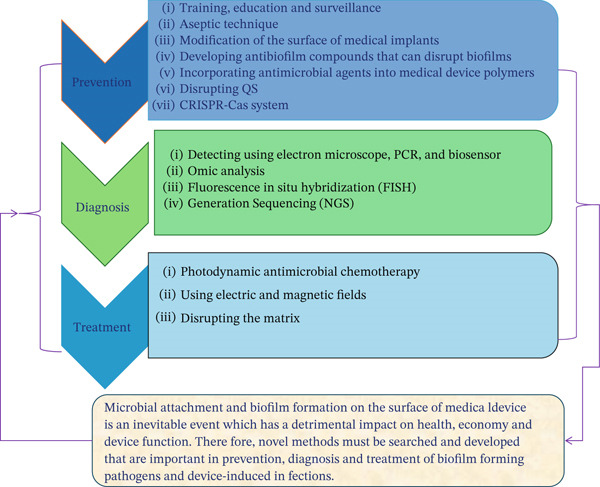
A glimpse of strategies being under investigation to overcome bacterial colonization on implantable devices [8, 13, 133].

Bacterial biofilm formation is a far‐reaching problem in the medical sector and a great challenge to circumvent, and with the emergence of antibiotic‐resistant strains, normal antibiotic therapy is ineffective [27, 45]. Treatment of biofilm‐induced implant infection requires long‐term treatment with high‐dose antibiotics [63] (Figure 4). Biofilm infections are not only difficult to treat, but also difficult to diagnose due to the difficulty of removing mature biofilms from the implant surface and their reduced growth of dormant bacteria within the biofilms [48]. Due to the development of antibiotic resistance in bacteria, there is an urgent need to discover new approaches to develop more effective therapeutics to treat MDR strains and biofilm‐associated infections [118]. Although it is a great challenge to thwart biofilm formation on medical devices, various preventive strategies have been designed (Figure 7). Strategies to prevent biofilm formation range from systematic approaches to control bacterial entry into sterile sites to localized biofilm suppression in medical devices [134]. Once biofilms are formed on the surfaces of biomaterials, they are difficult to eradicate so that preventive methods must be employed at their infant stage. For instance, surface modification of biomaterials, antimicrobial coatings, application of bacteriophage, immunotherapy, nanostructured coatings, photo irradiation, enzyme‐mediated approaches, QS quenchers, weak organic acid utilization, and alternative mechanical design have proven to be effective in reducing biofilm‐related infections by preventing the bacterial adhesion on medical devices [24, 58, 135] (Figure 7). As recent research works suggested, effective prevention of biofilm formation may be achieved by multifunctional surface coatings that provide both nonadhesive and antimicrobial properties imparted by antimicrobial peptides [136]. Surface coating with antibacterial and antibiofilm chemicals is a promising method to prevent planktonic cells from adhering to implant surfaces [8]. The use of polymer‐based medical devices which incorporate antimicrobial strategies is now becoming a promising approach used to reduce chronic infection and device failure [25, 58].

In order to prevent microbial colonization and subsequent infections, biomaterials endowed with antimicrobial surfaces are also recommended [137]. Biomaterials containing highly potent antimicrobials are considered the future remedies that will penetrate biofilms and eradicate the organisms [138] (Figure 7). Nowadays, antibiotic resistance is the biggest threat to global health. Therefore, alternative approaches must be searched to minimize its health consequences. For instance, the use of phages to inhibit bacterial infections is being followed as an alternative to antibiotics [139], and they are well suited as part of a multidimensional strategy to combat antibiotic resistance [140]. However, bacteria can also readily develop phage resistance [141]. Therefore, new strategies such as surface modification or coating are extensively explored that can prevent microbial attachment on medical surfaces instead of treating mature biofilms [33]. As indicated in Figure 7, besides surface coating, electric and electromagnetic fields are currently searched for treatment of bacterial colonization [8]. This strategy is gaining acceptance as the conventional approaches such as the use of phages, QS, and antimicrobial surfaces are frequently insufficient [142]. The usage of antiadhesion agents, antimicrobial peptides, QS inhibitors, aptamers, nanoparticles, and peptide nucleic acids represent a promising therapeutic approach in the treatment of biofilm‐induced infections. These antibiofilm agents are expected to intervene stages of biofilm development, including adhesion, intercellular polysaccharide adhesion, QS molecules, and cell–cell binding [139].

Along with the abovementioned strategies, nanomaterials are emerging as antibiofilm agents because of their great potential to combat and treat biofilm‐associated infections. For instance, carbon‐based nanomaterials have drawn attention in medical sectors [143], because many nanomaterials and nanosized carriers have the potential effect on bacterial diseases like reducing cell viability, interfering with the signaling molecule, and inhibiting/eliminating biofilms. Arrays of nanomaterials that range from metallic to polymeric nanoparticles have unique anti‐infective properties on microbial infections [144]. Metal and metal oxide nanoparticles offer a new option to target antibiotic resistance in gram‐negative bacteria [145]. For instance, a wide range of metal‐based nanoparticles such as zinc, zinc oxide, silver, gold, nitric oxide, aluminum, titanium oxide, and copper have shown antimicrobial activity [118] such as damaging bacterial membranes, resulting in indiscriminate antimicrobial activity on both gram‐positive and gram‐negative bacteria and fungi [80]. Along with nanoparticles, clustered regularly interspaced short palindromic repeats (CRISPR) are also offering promising avenues for fighting antimicrobial resistance by targeting and eliminating antibiotic‐resistant genes [129]. CRISPR‐Cas plays a prominent role in restricting horizontal gene transfer among bacteria and thus limiting the spread of resistance genes [146]. Notably, nanoparticle‐mediated CRISPR delivery offers enhanced effectiveness by improving cellular uptake, increasing target specificity, and enabling controlled release within biofilm environments [119].

QS is a cell density–dependent regulatory process that uses signaling molecules to control virulence gene expression and biofilm formation [145]. This complex intercellular communication system and the coordinated multicellular behavior of biofilm formation have been identified as promising targets for therapeutic and clinical management of microbial infections [147]. Targeting microbial QS‐associated virulence and biofilm development has proven to be the best approach to address the issue of antibiotic resistance [144]. Moreover, biofilm‐associated genes are also a potential target to thwart biofilm‐induced infections. Biofilm‐associated genes as potential molecular targets of nano‐Fe_3_O_4_ in *Candida albicans* may be a promising and an effective novel treatment strategy for biofilm‐associated infections [148].

Antimicrobial peptides as antibiofilm agents have received much attention in preventing device‐induced infection and biofilm formation. Antimicrobial peptides are small cationic molecules which have broad spectrum against microorganisms; especially, they exhibit strong antibiofilm properties against antibiotic‐resistant bacteria, which can be effective with different mechanisms at various phases of the biofilm development and on different molecular targets [139]. As research reports revealed melittin as a cationic antimicrobial peptide is effective against a wide range of bacterial pathogens alone, and offers a good synergistic activity in combination with some antibiotics against different MDR species pathogens [123]. Even if various preventive and therapeutic approaches are suggested, device‐induced infection and biofilm formation on medical devices become an enduring problem. Therefore, it is better to understand microbial attachment mechanisms and biofilm formation mechanisms to develop novel preventive and therapeutic approaches.

Effective management of biofilm‐associated infections demands not only innovative preventive and therapeutic measures but also substantial advancement in detection and diagnostic capabilities. Beyond their resistance to antimicrobial therapy and immune cells, biofilms are notoriously difficult to identify and diagnose. Detecting biofilm‐forming bacteria from clinical samples using conventional diagnostic methods frequently produces false‐negative results [36] which mislead medication and delayed treatments. One of the forefront problems that come along with this false result is inappropriate antibiotic prescriptions, which further worsen the health problem and contribute to the emergence of antimicrobial resistance. Due to the highly complex structure of biofilms and their inherent resistance to conventional detection techniques, detecting biofilm formation remains a major challenge in diagnostic microbiology [69]. Biofilm‐induced infections are mostly recurrent, persistent, and chronic, which are extremely difficult to diagnose [36]. Based on the conditions they encounter, bacteria can alternate between planktonic and sessile modes with significant morphological and physiological changes that further complicate the detection and diagnosis of biofilm‐associated infections and pathogens. Despite advances in biofilm research, there is no novel standardized protocol to detect biofilms in clinical settings [149, 150]. Moreover, lack of biofilm‐specific biomarkers and methods for nondestructive imaging are some of the challenges in the clinical area [151]. Especially, this issue could be challenging for developing nations where limited resources, inadequate laboratory infrastructure, and lack of specialized expertise are prevalent. As reported by Zakir et al. [152], laboratory services in developing countries often fail to meet minimum standards. Unless biofilm‐forming pathogens and associated infections are accurately detected and diagnosed, proper treatment is extremely challenging, leading to persistent and chronic infections which have a far‐reaching impact on patients′ health, disease surveillance, and economy. This persistent problem underscores the urgent need for advanced, sensitive, cost‐effective, and rapid diagnostic approaches capable of detecting biofilm‐associated infections at an early stage.

## 6. Conclusion

Indwelling medical devices are crucial in curing or ameliorating the agony of numerous people who have suffered in severe injuries. Although it is a big advancement in modern medicine, device‐induced infections become an intractable problem. This is because indwelling medical devices are prone to microbial colonization and biofilm formation which can lead to device‐induced infection, device malfunction, and removal from the patients which leads to economic loss. The pathogenesis of device‐associated infections is largely attributable to biofilm‐forming bacteria that show discrete characteristics with respect to growth rate, structural features, and protection from the host immune system and antimicrobial agents when compared with planktonic counterparts. These surface‐attached and structured microbial communities are recalcitrant to hostile conditions such as antibiotics and immune defense, posing challenges for detection and diagnosis. Biofilm‐induced infections are chronic and persistent, often resulting in treatment failure and recurrent infections, thereby posing significant challenges to public health and the economy. Device‐induced infection involves complex interactions between the pathogen, the biomaterial, and the host immune response. Thus, it is better to understand these interactions to develop both preventive and therapeutic approaches; especially the prevention of initial attachment is very important. Although arrays conventional and novel preventive and therapeutic approaches have been proposed, still medical devices are prone to bacterial attachment and biofilm formation on medical device surfaces. Generally, biofilm formation on medical devices is an inevitable event which has a stringent consequence on health, device function, and economy. Thus, the perpetual problem urges biomaterials industries, researchers, and healthcare professionals to explore alternative strategies aimed at mitigating the problem.

## Funding

No funding was received for this manuscript.

## Conflicts of Interest

The author declares no conflicts of interest.

## Data Availability

The data or information used to write this review article is available from the corresponding author upon request.
